# Moveluencer - movement promotion in the context of covid 19

**DOI:** 10.1093/eurpub/ckac131.334

**Published:** 2022-10-25

**Authors:** S Tuttner, P Holler, F Amort

**Affiliations:** Health- and Tourismmanagement, Universitiy of Applied Sciences - JOANNEUM, Bad Gleichenberg, Austria

## Abstract

In times of a pandemic, regular physical activity is recommended as protection against the additional stressors caused by a significantly changed daily routine and, above all, severe restrictions in social life. Within this context, promoting active mobility is a meaningful approach to improving the health of the population. The project ‘MOVEluencer’ is a physical activity promotion project following a multidimensional and participatory approach. It has been running since July 2021 in six small rural communities in Styria, a province of Austria (average: 2262 residents). These communities were selected based on inequality factors related to health and physical activity. The promotion of active mobility is at the heart of the project. The main target groups include children and adolescents, their parents, as well as older adults. The aim of the analysis phase was to identify infrastructural resources that are favorable to a physically active lifestyle through a photo contest. A total of 268 photos were sent in by 67 people (43% female). In spring 2022, ‘movement events’ were implemented to raise awareness in terms of active mobility among the population. These events were supplemented by the offer of fitness tests and approximately 265 persons (age: 10 to 84 years) participated. As a follow-on activity, 4 to 5 ‘walking buddies’ will be trained in each community and they will subsequently offer regular walking meetings. In the 3rd project phase, specific physical activity programs will be developed and implemented together with the target groups of the project. Aspects for the sustainable anchoring of physical activity promotion are considered in each project phase. It has been shown that awareness of the health benefits of active mobility in rural areas is rather low. Therefore, improving lifestyle and infrastructural conditions as well as measures to promote physical literacy are important.

**Key messages:**

• Awarenes among the population regarding the health benefits of active mobility is low in rural areas.

• Social media campaigns to promote physical activity are successful in all age groups.

